# Is it possible to objectify the visual pain scale?

**DOI:** 10.12669/pjms.316.8269

**Published:** 2015

**Authors:** Mehmet Ergin, Abdullah Sadik Girisgin, Zerrin Defne Dundar, Goknil Saniye Calik, Izzetin Ertas, Mehmet Taskin Egici

**Affiliations:** 1 Mehmet Ergin, MD, EP. Assistant Professor, Department of Emergency Medicine, Necmettin Erbakan University, Meram Medicine School, Konya, Turkey; 2 Abdullah Sadik Girisgin, MD, EP. Professor, Department of Emergency Medicine, Necmettin Erbakan University, Meram Medicine School, Konya, Turkey; 3 Zerrin Defne Dundar, MD, EP. Assistant Professor, Department of Emergency Medicine, Necmettin Erbakan University, Meram Medicine School, Konya, Turkey; 4 Goknil Saniye Calik, MD, EP. Konya Education and Research Hospital, Emergency Medicine Department, Konya, Turkey; 5 Izzetin Ertas, MD, EP. Resident Assistant, Department of Emergency Medicine, Necmettin Erbakan University, Meram Medicine School, Konya, Turkey; 6 Mehmet Taskin Egici, MD, EP. Resident Assistant, Emergency Medicine Department, Sisli Etfal Education and Research Hospital, Istanbul, Turkey

**Keywords:** Analgesia/pain control, Clinical management, Emergency department management, Renal colic

## Abstract

**Objectives:**

To test our hypothesis that a new modified VAS (mVAS) is superior and more objective than VAS in evaluating pain perception and treatment response between genders who have renal colic pain.

**Methods:**

The individuals in patient and control groups were first asked to mark the pain perceived during access of IV line (VAS_IV_ score). Then the patients with renal colic were asked to mark the pain they experienced before treatment (VAS_RC_ score) and at 15 and 30 minutes after the administration of the first analgesic drug. The modified VAS scores (mVAS score) were obtained by subtracting the VAS_IV_ score from VAS_RC_ score.

**Results:**

When VAS was used, the female patients had significantly higher level of pain at 0, 15, and 30^th^ minutes than men (p = 0.012, p = 0.001, and p = 0.003, respectively). However, there was not any significant difference at 0 and 30^th^ min between sexes while female patients had significantly higher level of pain scores only at 15^th^ minute according to mVAS scores (p = 0.027).

**Conclusion:**

We think that the mVAS is superior and more objective than VAS in evaluating pain perception and abolished the difference in the perceived level of pain due to gender.

## INTRODUCTION

Pain is an alarming symptom that develops in response to numerous clinical conditions and causes discomfort in patients. The incidence of patients with pain has been on the rise in recent years, and a greater number of patients have sought care in emergency departments (EDs) due to acute pain.^1^,^2^ One of the most important steps in the evaluation and treatment of underlying disease is palliating pain. Because pain is a subjective perception, scales such as the visual analog scale (VAS) or numeric rating scales have been developed in order to evaluate pain and responses to painkiller methods and drugs.^3^,^4^

The VAS is a scoring system that requires patient compliance describes the level of pain from zero to ten. It was claimed that the VAS is more useful in evaluating the effectiveness of pain treatment than in the evaluation of acute pain.^5^ Since the perception of pain differs from person to person, numerous studies have been conducted on various modified VAS systems in order to increase the objectivity of the VAS.^6^-^8^

Another major discussion point on pain perception is whether gender affects pain perception or not. While a majority of the studies support the assumption that women experience pain more frequently and more severely than men, others conclude that there is essentially no difference about the perception of pain and the response to pain treatment between the sexes.^2^,^9^,^10^ Moreover, recent studies report that the perception of pain is influenced by a variety of variables, such as genetics, socio-cultural factors, and analgesics chosen for treatment.^10^,^11^ However, bias created by the subjectivity of the pain scales used during those studies cannot be overcome.

In the present study, VAS was thought to be modified with threshold level by determining pain perceived during access of intravenous (IV) line. Our hypothesis is that a new modified VAS (mVAS) is superior and more objective than VAS in evaluating pain perception and treatment response between genders who have renal colic pain.

## METHODS

### Study Population

This single-center prospective clinical study was conducted on consenting adults (males and females aged 18 and older) who presented to Emergency Department (ED) of a university hospital and were diagnosed with and treated for renal colic pain between March 2010 and September 2011. The diagnosis of acute renal colic was made based on the findings of the patient history, physical examination, complete urinalysis, direct urinary system radiography, and if required, renal ultrasonography and non-contrast abdominal tomography. In order to standardize the study protocols, patients with acute pain other than renal colic were excluded from the study. The control group consisted of adult patients that presented to the ED for complaints other than pain. Approval for the study was obtained from the local ethics committee (ECN: 2011-111), and written consent was obtained from all patients enrolled in the study.

### Treatment Protocol

The same nurse performed access of IV line with a 20G catheter on the antecubital regions of individuals on the patient and control groups. In patient group, hydration with 500 mL of a 0.9% NaCl solution was provided together with a single dose of a non-steroid anti-inflammatory drug (NSAID) delivered intravenously. An additional dose of the same agent or a different type of analgesic was given 30 minutes after the initial dose of the IV NSAID to patients for whom an adequate analgesia could not be achieved. The patients on the control group, who presented to the ED for complaints other than acute pain, were also started on IV lines using the same size catheters and with the same nurse.

### Study Protocols

A 100-mm VAS (0 = no pain, 100 = worst pain) was used to assess the pain perceived by the study groups. Those patients in both groups were first asked to mark the pain perceived during access of IV line (VAS_IV_ score) on the VAS card. Then the patients with renal colic were asked to mark the pain they experienced before treatment (VAS_RC_ score) on the VAS card. Finally, the patients marked their pain levels at 15 and 30 minutes after the administration of the first analgesic drug. The patients of the control group were just asked to mark their VAS_IV_ scores on the VAS cards.

The pain levels marked as VAS_IV_ score were assumed as the standard to establish the patients’ pain thresholds. The modified VAS scores (mVAS score) were obtained by subtracting the VAS_IV_ score from VAS_RC_ score, and were considered the true indication of pain perception:

[mVAS score = VAS_RC_ score - VAS_IV_ score]

We evaluated the differences for the pain perceived during access of IV line between patient and control group. Further, we grouped the patients based on gender and evaluated the changes in VAS and mVAS scores over time in order to evaluate differences between sexes.

### Statistical Analyses

All data were evaluated using SPSS (version 16.0, SPSS Inc., Chicago, IL). The data were presented as the median (minimum (min) – maximum (max)), mean and standard deviation. Differences in VAS scores between groups were tested using the Mann Whitney U and independent two-sample *t*-tests. Variations in VAS scores over time were tested using Dependent two-sample *t*-tests and Wilcoxon tests.

## RESULTS

A total of 96 patients were included in the study. Of those, 63 (65.6%) were in the patient group and 33 (34.3%) were in the control group. The average age of the patient group was 37.8±13.8, while it was 49.8±20.4 for the control group. Forty-two members (66.7%) of the patient group were males, whereas 16 members (48.5%) of the control group were males.

**Fig.1:**
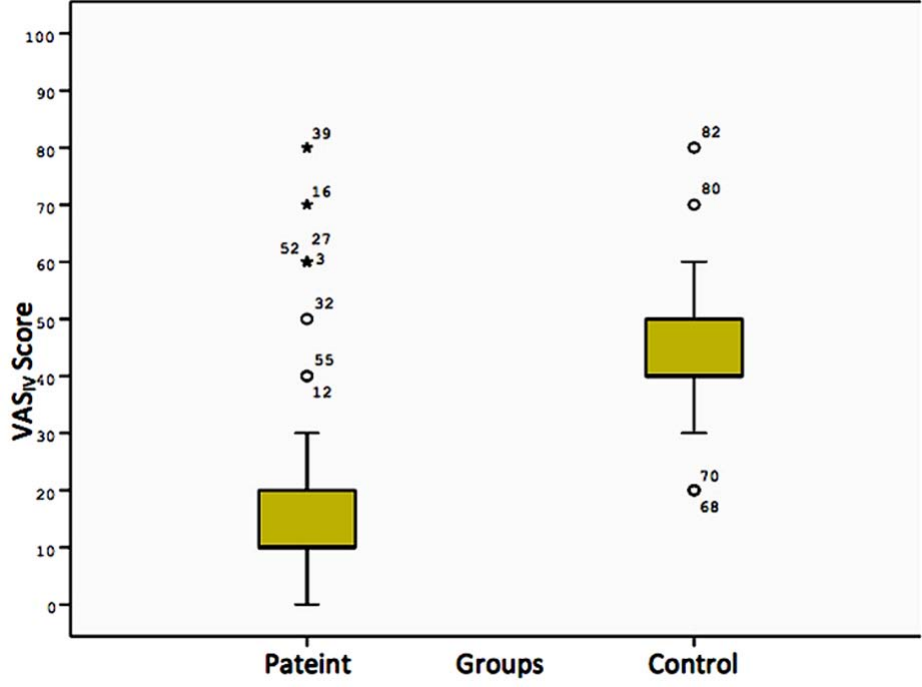
Comparison of VAS_IV_ scores in the patient and control groups*.

**Fig.2:**
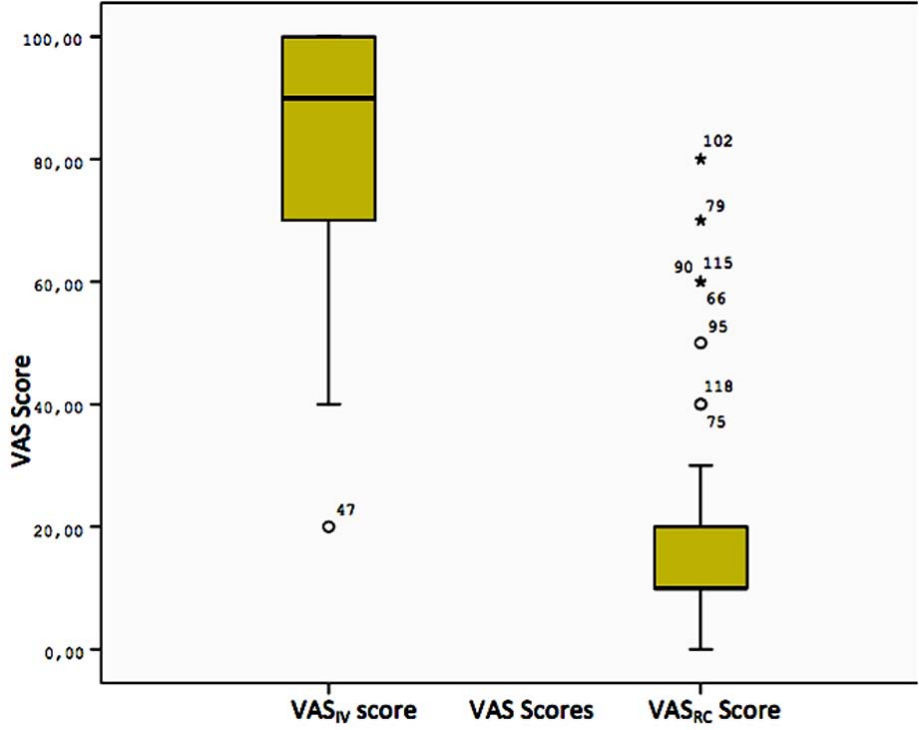
Comparison of VAS_IV_ and VAS_RC_ scores in the patient group*.

When VAS_IV_ score is taken into account, the patient group reported significantly less pain during access of IV line than did the control group (p < 0.001) (Table-I, Fig.1). When we compare VAS_IV_ score and VAS_RC_ score in the patient group, the level of pain described during access of IV line was significantly less than pain due to the acute renal colic (p < 0.001) (Table-II, Fig.2).

**Table-I:** Comparison of VAS_IV_ scores in the patient and control groups.

	N	Min	Median	Max	Mean	Std. Deviation	Variance
Patient	63	0.00	10.00	80.00	19.04	17.57	308.75
Control	33	20.00	40.00	80.00	44.24	13.46	181.43
Test statistics		Mann-Whitney U = 245.500				k* = 28.154, z = –0.335	
p-value		<0.001				0.368	

* Klotz nonparametric test for scale

VAS_IV_: the level of pain during access of IV line.

**Table-II:** Comparison of VAS_IV_ and VAS_RC_ scores in the patient group.

	N	Min	Median	Max	Mean	Std. Deviation	Variance
VAS_IV_ score	63	0.00	10.00	80.00	19.04	17.57	308.75
VAS_RC_ score	63	20.00	90.00	100.00	82.53	19.99	399.89
Test statistics		Wilcoxon Z = –6.749				k* = 58.984, z = 0.007	
p-value		<0.001				0.502	

* Klotz nonparametric test for scale

VAS_IV_: the level of pain during access of IV line; VAS_RC_: the level of pain due to renal colic.

**Fig.3:**
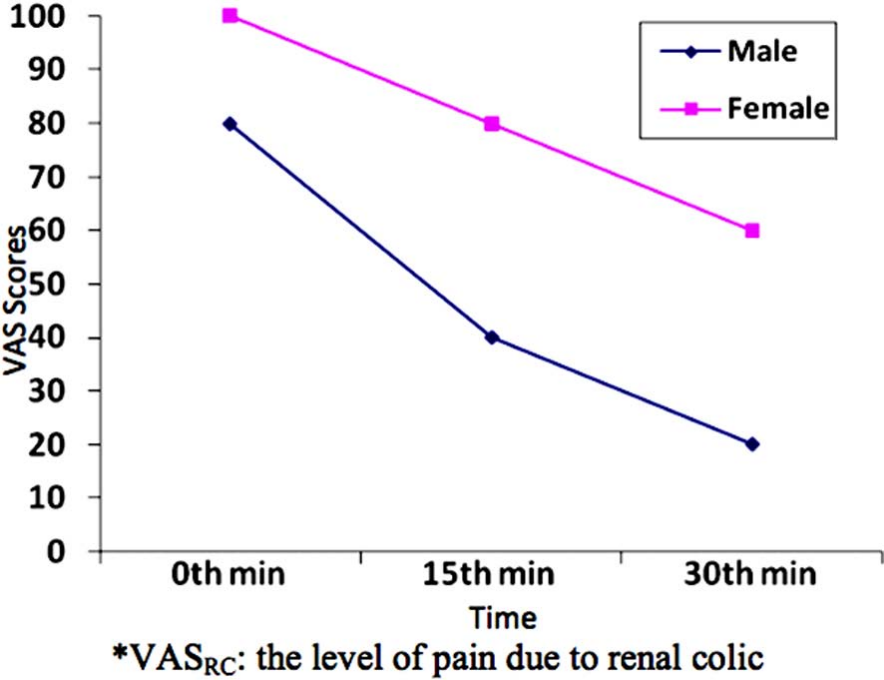
The changes in the VAS_RC_ scores for patient group at 0, 15 and 30^th^ min*.

When we evaluate changes in VAS_RC_ score over time according to gender, the female patients had significantly higher level of pain at 0, 15, and 30^th^ minutes than men (p = 0.012, p = 0.001, and p = 0.003, respectively) (Table-III, Fig.3).

**Table-III:** Comparison of the patient group between sexes for VAS_RC_ scores at 0, 15 and 30.min.

Time	Groups	N	Min	Median	Max	Mean	Std. Dev.	Tests	p-value
0. min	Female	21	50.00	100.00	100.00	90.47	15.64	z = –2,516*	0.012
	Male	42	20.00	80.00	100.00	78.80	19.90		
15. min	Female	21	30.00	80.00	100.00	73.33	23.09	t = 3.614**	0.001
	Male	42	0.00	40.00	100.00	47.61	28.18		
30. min	Female	21	10.00	60.00	100.00	55.71	30.26	z = –2.977*	0.003
	Male	42	0.00	20.00	100.00	30.95	32.74		

* Mann-Whitney U test is used,

** Independent two-sample t-test is used

VAS_RC_: the level of pain due to renal colic.

**Fig.4:**
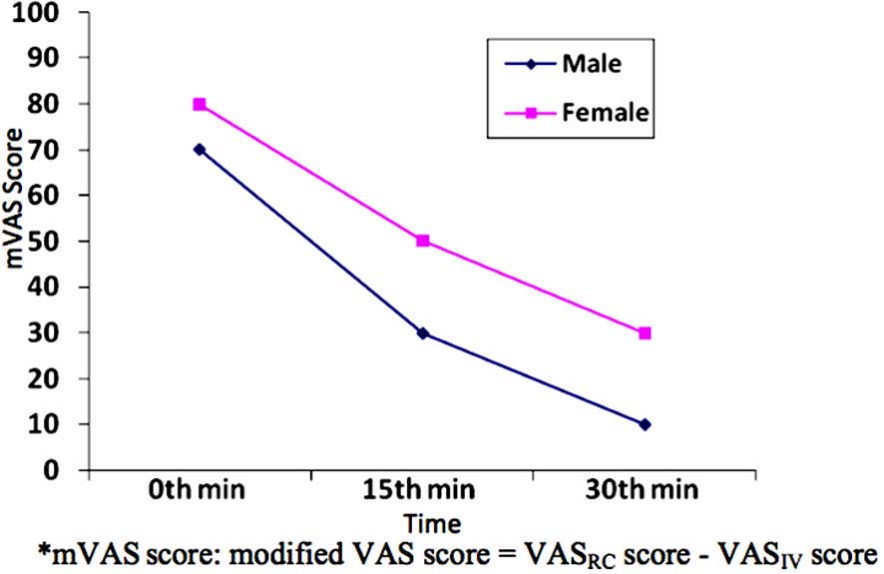
The changes in the mVAS scores for patient group at 0, 15 and 30^th^ min*.

When we consider changes in mVAS score over time according to gender, there was not any significant difference at 0 and 30^th^ min between sexes while female patients had significantly higher level of pain only at 15^th^ minute (p = 0.027) (Table-IV, Fig.4).

**Table-IV:** Comparison of mVAS scores between sexes in the patient group.

Time	Groups	N	Min	Median	Max	Mean	Std. Dev.	Tests	p-value
0. min	Female	21	0.00	80.00	100.00	68.09	23.79	z = –0.885*	0.376
	Male	42	–10.00	70.00	100.00	61.42	28.41		
15. min	Female	21	–20.00	50.00	100.00	50.00	30.16	t = 2.260**	0.027
	Male	42	–60.00	30.00	90.00	29.76	35.02		
30. min	Female	21	–50.00	30.00	80.00	31.42	36.64	t = 1.676**	0.099
	Male	42	–60.00	10.00	90.00	14.04	39.82		

* Mann-Whitney U test is used,

** Independent two-sample t-test is used

mVAS score: modified VAS score = VAS_RC_ score - VAS_IV_ score.

## DISCUSSION

With this study, we wanted to show that modified VAS method is more objective in evaluating pain perception and eliminates gender differences in pain perception. In order to find the ideal method in our study, we assumed that different scores reported by individuals after the standard procedure like accessing IV line were actually the same. By aligning the different scores reported for standard procedure on the same line, we aimed to determine the actual level of pain perceived by the patients experiencing more severe pain. We think that such an adjustment is a simple yet valuable method to standardize and establish the pain threshold of an individual.

Pain is a subjective finding. Even though people perceive same level of pain, their expression of pain can be different among individuals. Ideally, clinicians wish to have a method that overcomes differences in expression so that the perception of pain can be described more objectively. Research has been conducted for years for the purpose of developing an ideal pain expression method. VAS has been reported as a simple yet effective methodology for the assessment of acute and chronic pain in the studies.^5^,^12^,^13^ However, recent studies that affirmed the shortcomings of VAS in expressing perceived pain have emerged in the literature.^14^,^15^ The first modification performed to eliminate the subjectivity in the evaluation of pain based on differences in perception was the numeric version of VAS, called the *numeric rating scale*.^4^,^16^ Another recent study evaluated whether the horizontal or vertical layout of the VAS scale has an effect on the expression of perceived pain. It was found that vertical scales are as efficient as the horizontal scales.^7^ The VAS scale was criticized as being a single-dimensional tool that was inefficient in evaluating chronic pain. Variables such as the duration of pain and the frequency of painful episodes were then added to overcome these difficulties.^6^ However, none of these modifications addressed the differences in the perception and expression of pain among individuals and between genders.

Patients with renal colic pain expressed significantly less pain during acess of IV line than the control group (Table-I, Fig.1). Studies report that more severe pain can mask less severe pain.^17^ The results of our study supported these findings. Even though more severe pain masks lesser pain, standardization of VAS using threshold level of pain induced by a standardized procedure will allow clinicians to evaluate the pain more objectively.

There are other deterministic factors of perceived and expressed acute pain than the pain threshold. There are numerous studies in the literature that report differences in pain perception and responses to analgesic treatment between genders.^1^,^17^,^18^ While it has been shown that these differences are based on multiple factors, including psychosocial, cultural, neurophysiological, and genetic factors, the results of these studies fail to represent an agreement.^2^,^19^-^21^ Despite the generally accepted statement that women perceive pain more severely and have poorer responses to analgesic treatment, there are studies in the literature indicating no significant difference in pain perception between genders.^2^,^22^ In our study, we found that, when evaluated with the VAS_RC_ scores, women perceived renal colic pain more severely than men, and that they got less relief from analgesic treatments after 15 and 30 minutes (Table-III, Fig.3). However, when we used mVAS scores, we found no significant difference in pain perception between genders before treatment and 30 minutes after treatment (Table-IV, Fig.4). Men reported greater relief after 15 minutes of treatment according to mVAS scores than women in our study. We think that our modified VAS method abolished the difference in the perceived level of pain due to gender. Our results also support the findings that men’s responses to analgesics are faster than women.

VAS is a single-dimensional pain evaluation method that does not prevent subjectivity. When VAS is modified by pain stimulated after a standard procedure – it becomes a more realistic and usable method of expressing the actual level of pain perceived by individuals. Alternative methods of standardized procedure for stimulating pain (e.g. devices like electrical or pin pain stimulators) in order to objectify the expression of pain, as we did in our study, are needed to optimize the VAS scale. Furthermore, asking patients on whom the VAS scale was used to quantify a previous pain experience on the VAS scale may further assist in optimizing the objectivity of the VAS scale.

### Limitations of the Study

This was a single center study with a limited number of patients. The accuracy of mVAS also should be tested on cases with different racial groups and different source of acute pain. The previous experience of patients about pain was not considered.
